# RETRACTION: Dynamic Protective Effect of Chinese Herbal Prescription, Yiqi Jiedu Decoction, on Testis in Mice with Acute Radiation Injury

**DOI:** 10.1155/ecam/9807323

**Published:** 2025-09-23

**Authors:** Evidence-Based Complementary and Alternative Medicine

RETRACTION: T. Wang, X. Zhang, and J. Yang, “Dynamic Protective Effect of Chinese Herbal Prescription, Yiqi Jiedu Decoction, on Testis in Mice with Acute Radiation Injury,” *Evidence-Based Complementary and Alternative Medicine* 2021 (2021): 6644093, https://doi.org/10.1155/2021/6644093.

The above article, published online on 02 June 2021 in Wiley Online Library (https://wileyonlinelibrary.com), has been retracted by agreement between the authors and John Wiley & Sons Ltd. The retraction has been agreed due to concerns and faults in the experimental design as identified by the authors. These concerns invalidate the conclusions.

